# Citramalic acid and salicylic acid in sugar beet root exudates solubilize soil phosphorus

**DOI:** 10.1186/1471-2229-11-121

**Published:** 2011-08-26

**Authors:** Reza Khorassani, Ursula Hettwer, Astrid Ratzinger, Bernd Steingrobe, Petr Karlovsky, Norbert Claassen

**Affiliations:** 1 Department of Crop Science, Plant Nutrition, Georg-August-University Göttingen, Carl-Sprengel-Weg 1, D-37075 Goettingen, Germany; 2 Department of Soil Science, Faculty of Agriculture, Ferdowsi University of Mashhad, Iran; 3 Department of Crop Science, Molecular Phytopathology and Mycotoxin Research, Georg-August-University Göttingen, Grisebachstrasse 6, D-37077 Goettingen, Germany

## Abstract

**Background:**

In soils with a low phosphorus (P) supply, sugar beet is known to intake more P than other species such as maize, wheat, or groundnut. We hypothesized that organic compounds exuded by sugar beet roots solubilize soil P and that this exudation is stimulated by P starvation.

**Results:**

Root exudates were collected from plants grown in hydroponics under low- and high-P availability. Exudate components were separated by HPLC, ionized by electrospray, and detected by mass spectrometry in the range of mass-to-charge ratio (m/z) from 100 to 1000. Eight mass spectrometric signals were enhanced at least 5-fold by low P availability at all harvest times. Among these signals, negative ions with an m/z of 137 and 147 were shown to originate from salicylic acid and citramalic acid. The ability of both compounds to mobilize soil P was demonstrated by incubation of pure substances with Oxisol soil fertilized with calcium phosphate.

**Conclusions:**

Root exudates of sugar beet contain salicylic acid and citramalic acid, the latter of which has rarely been detected in plants so far. Both metabolites solubilize soil P and their exudation by roots is stimulated by P deficiency. These results provide the first assignment of a biological function to citramalic acid of plant origin.

## Background

Sugar beet and wheat are similar in their phosphorus (P) efficiency with regard to shoot production [1] but they appear to use different mechanisms to overcome low availability of soil P. Wheat has a large root system that compensates for low P influx when P availability is low, whereas sugar beet is able to achieve high P influx despite low P availability in soil [1]. The higher P influx of sugar beet compared to other plant species cannot solely be due to a more efficient uptake physiology. At low P availability, soil P transport is the limiting factor in P uptake [2]. Hence, the high P influx of sugar beet is attributed to the ability of the plant to mobilize, i.e. solubilize, P in the soil. This mobilization is most likely due to chemical modification of the rhizosphere by root exudates.

Plants exude up to 30% of assimilated carbon into the rhizosphere [3-5]. The composition of root exudates is complex and includes high molecular weight (HMW) and low molecular weight (LMW) molecules. HMW exudates include secreted enzymes and mucilage, which consists mainly of polysaccharides. Liebersbach et al. [6] showed that HMW exudates can increase P availability for plants, probably because carboxyl groups of polysaccharides interact with P-binding sites in the soil, which releases P into the soil solution. The long chains of polygalacturonate in HMW exudates may also cover soil particles and reduce the re-adsorption of phosphate [7]. Furthermore, the ability of HMW exudates to swell and absorb water may facilitate P diffusion toward the root [8]. LMW exudates include organic acids, sugars, phenolics, amino acids, phytosiderophores, flavonoids, vitamins and other compounds [4,5,9]. Phenolics might affect the speciation of iron (Fe) by complexation and thus might increase the availability of P occluded by Fe-oxides [10]. Organic acids, especially citrate, malate, and oxalate, are the root exudates most frequently investigated with regard to P mobilization.

P deficiency usually increases the root exudation rate and alters the composition of exudates [11,6], which results in an enhanced release of organic acids into the soil [12,13]. Hernandez [14] showed that the amount of organic acids was less in P-stressed roots than in P-sufficient roots of common bean; the reduced amount of organic acids in the P-deficient roots likely reflected exudation of organic acids from the root into the rhizosphere. These observations suggest that organic acids are involved in P acquisition.

Different mechanisms have been proposed for the facilitation of P acquisition by organic acids. For instance, citrate might increase the availability of P in the soil by binding calcium (Ca) and thus reducing the formation of insoluble complexes of Ca with P [15]. Citrate may also replace phosphate in complexes with Fe- and Al-oxides/hydroxides (ligand exchange). These processes release P into the soil solution as shown by Gerke et al. [16], who described a positive relation between citrate sorption and P-solution concentration. Furthermore, citrate can complex Fe, forming a citrate-Fe-P polymer that is soluble and can diffuse to the roots. The root reduces the Fe, thus breaking the polymer and releasing P directly at the root surface [17]. Hence, in soil incubation experiments with different organic acids, citrate often had the largest effect on P release.

It is still not clear, however, whether the beneficial effect of citrate and other organic acids on P availability actually occur in the rhizosphere. Most soil incubation experiments investigating the P-releasing effect of citrate were performed at relatively high concentrations of citrate (> 1 mmol L^-1^) while the amount of organic acids released by roots is relatively low. Furthermore, organic acids are rapidly decomposed by rhizosphere microorganisms and adsorbed to soil particles. Because the concentration of citrate in the rhizosphere soil solution is very low (typically < 10 μM; [18]), the contribution of citrate to P release remains questionable. An exception is the rhizosphere of cluster roots, which have much greater root surface area and exudation rates than non-clustered roots and which generate a low pH in the rhizosphere and therefore support only a low level of microbial activity [19,20]. Under these conditions, citrate might be a key factor for increasing the availability of P for plants.

For plants without cluster roots, i.e., for most plant species, other root exudate compounds, in place of or together with citrate, may play important roles in increasing P availability. For example, the high P-uptake efficiency of pigeon pea is due to the exudation of piscidic acid [21], which is exuded in lower amounts than citrate but releases P more efficiently. So far, only pigeon pea is known to counteract P deficiency by exuding piscidic acid. Besides common organic acids, as yet unknown components of root exudates may facilitate acquisition of soil P by plants. Such metabolites may be identified by comparing the composition of root exudates produced under low and high P supply. The use of HPLC coupled with electrospray ionization and a mass spectrometer (HPLC-ESI-MS) is particularly suitable for this purpose because it allows for simultaneous untargeted detection of a large number of substances with concomitant determination of their molecular weight.

The objective of this study was to use HPLC-ESI-MS to identify components of exudates of sugar beet roots that might increase P availability in the soil. Assuming that P-deficient plants would exude larger amounts of the compounds that solubilize soil P, root exudates were collected from sugar beet plants grown in nutrient solution with sufficient and with low P supply. Components of root exudates produced in larger amounts under low P supply were identified by differential metabolic profiling and were tested for their ability to solubilize P in soil.

## Results

### Shoot dry weight and shoot P concentration

**Figure 1:**
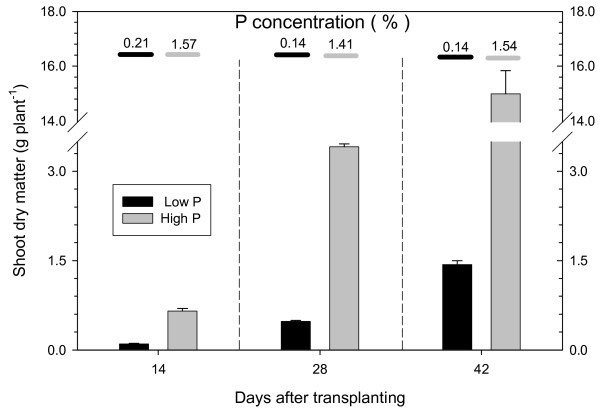
**Dependance of shoot dry weight and P content on P supply**. Shoot dry matter and P concentration of sugar beet grown in nutrient solution containing initially 2 (low P) or 500 (high P) μM P is shown. Values from three harvest times are given as means ± standard error of 3 replicates.

Sugar beet was grown in nutrient solution with 2 and 500 μmol P L^-1^. The low P concentration resulted in a decreased yield that was only 10-15% of the yield achieved with the high P concentration (Figure 1). In the low P treatment, P concentration in shoot dry matter was 0.14-0.21%, which is much less than the range considered sufficient (0.35-1.10%, [22]), indicating that the plants grown under low P were P deficient. The high-P plants achieved P concentrations of about 1.5% in dry matter (Figure 1).

### Exudation rate

**Figure 2:**
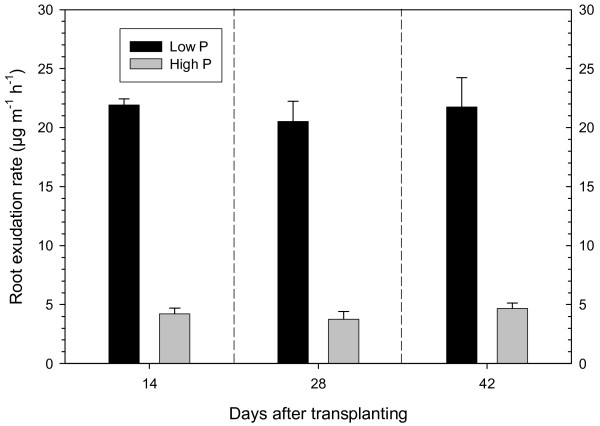
**Root exudation rates**. Rate of root exudation of sugar beet grown at low (2 μM) and high (500 μM) P is shown. Values originate from three harvest times and are given as means ± standard error of 3 replicates.

At each harvest, root exudates were collected in a trap solution over a 2 h period. The trap solution was lyophilized, and the dry matter was weighed and was considered to indicate the quantity of root exudates. The exudation rate was 4-5 times greater in the P-deficient plants than in the well-supplied plants at all three harvests (Figure 2). The exudation rate remained constant over the whole growing period. A high exudation rate under P deficiency--especially a high exudation of carboxylates--has often been shown [13,16,23]; see also review of Jones [18]. The focus of this study was the difference in the composition of the exudates between low-P and high-P plants.

### HPLC-MS analysis of root exudates

Full-scan HPLC-MS data were collected on root exudates as described in Methods section. The signals were normalized to compare low-P with high-P samples separately for each harvest. The normalized data were manually inspected to remove signals originating from solvent adducts and isotope peaks. Background peaks were removed that occurred in the controls. The profiles of high-P and low-P exudates were compared, and a total of 55 and 12 signals were detected in a negative and positive ionization mode, respectively, that were at least 5-times higher in low-P exudates than in high-P exudates (Table 1). Most of these signals, however, occurred only at a single harvest. Only 14 signals in negative mode were higher in exudates collected under low-P conditions than under high-P conditions in at least two harvests. Among these, ions [M-H]^- ^with m/z of 137 and 147 were selected for further investigation because of their distinct MS signals and the high reproducibility of the signals among the harvests.

**Table 1:** Number of HPLC-MS signals in sugar beet root exudates enhanced under low-P conditions

Harvest	Negative ionization (m/z 100-400)	Negative ionization (m/z 400-1000)	Positive ionization (m/z 100-1000)
1^st^	35	1	1

2^nd^	12	8	7

3^rd^	15	4	6

The criterion for the inclusion of a signal in the list was a threshold of at least 5-times enhancement of signal intensity after normalization for low-P as compared to high-P conditions.

### Phosphorus mobilization in soil

**Figure 3:**
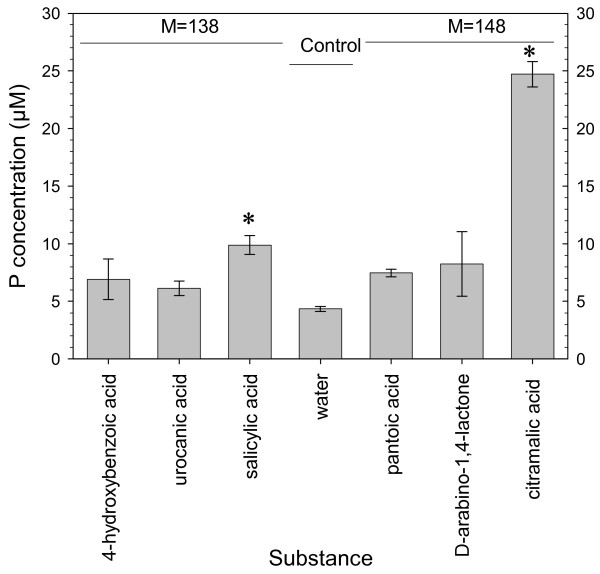
**Solubilization of soil P**. Effect of different substances on P concentration in the solution of soil suspension (8 g soil in 40 mL of distilled water with tested substances at 1 mmol L^-1^) after 5 h of incubation is shown. Values are given as means ± standard error of 3 replicates.

Candidate compounds were tested for the ability to solubilize P from soil. Six compounds were selected from the KEGG database [24] based on their molecular mass, the presence of carboxylic acids and commercial availability: 4-hydroxbenzoic acid, urocanic acid, salicylic acid, pantoic acid, D-arabino-1,4-lactone, and citramalic acid. Compounds with carboxylic acid groups were selected because the effect of organic anions on the mobilization of phosphate in soil is mediated by their functional groups [25], among which carboxyls are known to play a central role [26]. The results of P solubilization experiment are shown in Figure 3. Only salicylic and citramalic acid (molecular weight 138 and 148, respectively) increased P concentration significantly compared to the water control. The increase in concentration was by a factor of 2 and 6, respectively. The use of 1 mmol L^-1 ^solutions of candidate compounds in a ratio of 5 mL g^-1 ^of soil resulted in the addition of 5 μmol of compounds per g of soil. This quantity was based on the exudation rate of 22 μg m^-1^h^-1^, and by assuming that this rate was maintained for 7 h, that the radius of the rhizosphere was 0.5 mm, and that the exudate had a molecular weight of 147. The calculated value was increased by a factor of 5, which corresponds to a higher exudation rate than actually measured. However, local concentration of root exudates is higher than the concentration in bulk soil solution. High concentrations of the candidates for P-solubilizing metabolites were also used to generate prominent, unequivocal effects. Fox et al. [27] used the same concentration (1 mmol L^-1^) and the same soil-solution ratio (1:5) to compare the ability of organic acids to mobilize P in a Spodic Horizon.

### Identification of salicylic and citramalic acids in root exudates of sugar beet

**Figure 4:**
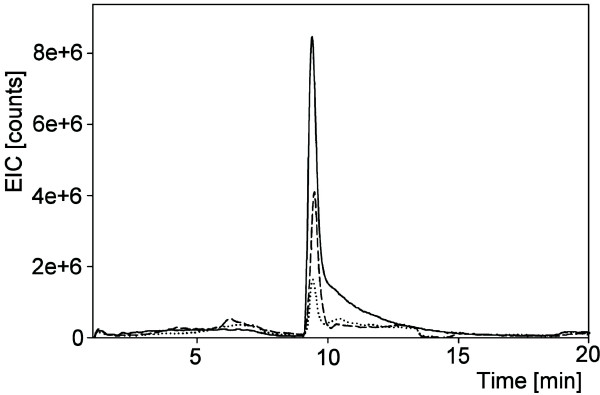
**Stimulation of citramalic acid exudation by low P availability**. Extracted ion chromatogram from HILIC-HPLC analysis of citramalic acid standard (100 ng/mL) (solid line), root exudates generated under low-P conditions (dashed line), and root exudates generated under high-P conditions (dotted line). Mass transition 147 > 87 after negative ionization was recorded.

**Figure 5:**
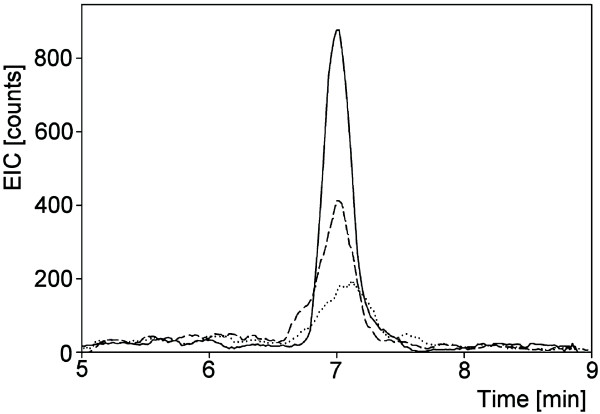
**Stimulation of salicylic acid exudation by low P availability**. Extracted ion chromatogram from RP-HPLC analysis of salicylic acid standard (100 ng/ml) (solid line), root exudates generated under low-P conditions (dashed line), and root exudates (dotted line) generated under high-P conditions. Mass transition 137 > 93 after negative ionization was recorded.

The mass spectrometric signals originating from root exudate components with putative molecular weights 138 and 148 were compared with the signals generated by pure salicylic and citramalic acid standards, respectively. For this comparison, samples originating from the same level of P supply for all three harvests were pooled to give a high-P pool and a low-P pool. A comparison of retention times and fragmentation patterns, based on reverse phase chromatography for salicylic acid and HILIC for citramalic acid (see Methods section), proved that the root exudate signal with an m/z value of 137 [M-H]^- ^originated from salicylic acid and that the signal with an m/z value of 147 [M-H]^- ^originated from citramalic acid. Salicylic and citramalic acids were present in root exudates originating from both low- and high-P treatments but concentrations of the two compounds were higher with the low-P than high-P treatment (Figures 4 and 5).

## Discussion

Root exudates were collected from roots grown in nutrient solution which may differ from soil grown roots by, for example, the lack of root hairs. However, dense root hairs are formed in nutrient solution under conditions of P deficiency [28]; actually the effect of P deficiency on root hairs has often been studied on hydroponically grown roots [29]. It therefore appears safe to assume that the root exudation patterns caused by different P supply are comparable between solution and soil grown plants.

**Figure 6:**
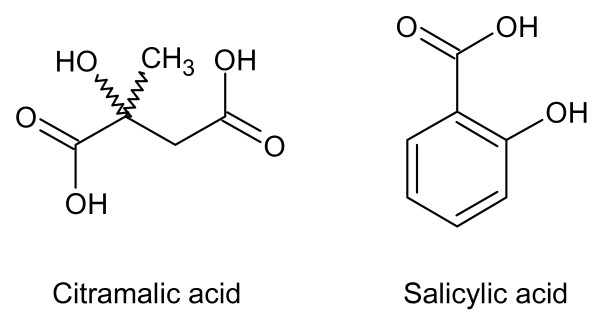
**Structures of citramalic acid and salicylic acid**.

The ability of salicylic and citramalic acid to mobilize P from soil is related to their carboxyl and hydroxyl functionalities (Figure 6). The effect of organic anions on the mobilization of phosphate from soils and metal oxides increases with the number of carboxylic groups [26]. The relative position of hydroxyl and carboxyl groups is important [21] because it controls the ability of the substance to chelate Fe ions in soils where P is associated with Fe.

The effect of salicylic acid on P availability in soil has been studied by Staunton and Leprice [30], who showed that salicylate increased the P concentration in the soil solution of a neutral calcic Luvisol. The competition between salicylic acid and P for sorption sites in two variable-charge soils was documented as well by Xu et al. [31]. On the other hand, Fox et al. [27] compared 16 organic acids and found no significant differences between salicylic acid and water in P solubilization. The discrepancy between their results and Staunton and Leprice [30], Xu et al. [31], and this study was likely caused by differences in the chemical form of P in the different soils.

Citramalic acid is known as a bacterial metabolite and an important chiral building block for synthetic pharmaceuticals, but it has rarely been detected in plants. Only three reports of citramalic acid in plants exist. It was found in apple peels 57 years ago [32], it was detected in tomato juice [33], and it was identified as one of uncommon metabolites during metabolic profiling of *Arabidopsis thaliana *[34]. Neither the biosynthetic pathway nor the physiological function of citramalic acids is known in plants. Our results provide the first putative assignment of a biological role to this uncommon plant metabolite.

We identified two among the eight mass spectrometric signals that were consistently enhanced in sugar beet root exudates under low-P conditions. Further work is needed to identify the remaining compounds and assess their ability to solubilize soil P.

## Conclusions

The enhanced exudation of citramalic acid and salicylic acid by sugar beet roots under low P availability and solubilization of soil P by these metabolites strongly indicate that the function of citramalic acid and salicylic acid in root exudates is to increase the availability of P. While the presence of salicylic acid in root exudates and its function is established, citramalic acid has rarely been observed in plants before. Solubilization of soil P is the first biological role assigned to citramalic acid in plants.

## Methods

### Hydroponic experiment

Sugar beet plants were grown in hydroponic culture in a growth chamber. Sugar beet seeds were sown in sand and grown only with distilled water for 14 days. Seedlings of similar size were selected, washed free of sand with distilled water, and carefully transferred into pots containing 12 L of aerated nutrient solution. The experiment included two levels of P, three harvests dates, and three replicate pots for each combination of P level and harvest date. Plants were grown in a growth chamber at 16/8 h light/dark cycle, 25°C/16°C day/night temperature, 60%/75% relative humidity, and 41 W m^-2 ^PAR (photosynthetically active radiation during the day time). The composition of the nutrient solution was 1 mM Ca(NO_3_)_2_.4H_2_O, 0.1 mM NH_4_NO_3_, 0.2 mM KCl, 0.1 mM MgSO_4_.7H_2_O, 17.9 μM Fe-EDTA, 16 μM H_3_BO_3_, 9.1 μM MnCl_2_.4H_2_O, 0.8 μM ZnSO_4_.7H_2_O, 0.5 μM (NH_4_)_6_Mo_7_O_24_.4H_2_O, 0.3 μM CuSO_4_.5H_2_O. Phosphorus was added as NaH_2_PO_4 _in the concentration of 2 or 500 μM, designated as low-P and high-P, respectively. The number of seedlings per pot depended on the planned harvest date for that pot: 15, 10, and 6 plants were placed in pots intended for the first, second, and third harvest, respectively. Plants were harvested every 2 weeks after transplanting. The nutrient solution was replaced and pH was measured every second day. At each harvest, root exudates were collected (see next section), shoot dry weight was recorded, and the shoot P concentration was determined after digestion of 0.3 g of finely ground shoot material in 4 mL of HNO_3 _and 2 mL of H_2_O_2 _under pressure at 175°C for 1 h in a microwave oven. P was determined by the molybdate-vanadate method of Scheffer and Pajenkamp [35]. Root length was determined with the line-intercept method [36].

### Root exudate sampling

Whole root systems of intact plants were carefully washed with running de-ionized water to remove the nutrient solution. For collection of exudates, the whole root system was dipped into aerated de-ionized water in a glass container, the volume of which depended on the size (age) of the root system. The container was covered with aluminium foil to create dark conditions for roots. The roots were kept in the water for 2 h under the same controlled climate conditions described for plant growth. Röhmheld and Neumann [23] showed that for a short exudation period of 2 h, the roots were not harmed by the de-ionized water and no significant microbial degradation of the exudates occurred [23]. The collected root exudates were immediately frozen at -30°C, freeze dried, and weighed.

### Preparation of samples and standards for HPLC-MS analysis

A 0.5-mg sample of each freeze-dried root exudate was weighed into an HPLC vial, and 100 μL of acetonitrile was added. After 30 min, samples were vigorously shaken. Then 900 μL of HPLC-quality water was added, and the samples were shaken again and filtered through Teflon membrane filters (0.2-μm pore size, 13 mm diameter, Optiflow-TF, Wicom Germany GmbH, Heppenheim, Germany). Standards of citramalic acid and salicylic acid were prepared as 10 mg/mL stock solutions in methanol:water (50:50 v/v) and diluted to 100 ng/mL in mobile phase used for column equilibration.

### Metabolic profiling of root exudates

For metabolic profiling of root exudates, an HPLC system coupled to an ion trap mass spectrometer was used. HPLC separation was carried out as described [37]. Mass spectrometry was performed with the 500-MS LC ion trap equipped with an electrospray ion source (Varian, Darmstadt, Germany). Nebulizer and drying gas pressures were set to 50 and 20 psi (345 and 138 kPa), respectively. Drying gas temperature was set to 350°C at the beginning of the gradient and was reduced gradually to 250°C with the increasing proportion of methanol in the eluate. For the detection of positive and negative ions, needle voltages were set to 5000 and -3500 V and shield voltages to 600 and -600 V, respectively. The capillary voltage was +/-50 V. In positive ionization mode, ions with an m/z of 100 to 1000 were collected in a single run, while in negative mode ranges of m/z 50-400 and m/z 400-1000 were scanned separately. The scan rate was 5000 Da/s, and three consecutive scans were averaged. MS data were transformed into chromatograms using MS Data Review 6.9 (Varian) and converted into netCDF format. Data from positive and negative ionization modes were separately processed as follows: minor differences in retention times were corrected by peak alignment performed with XCMS version 1.5.2 [38] run under R package 2.4.0. The resulting data were normalized to compensate for differences caused by uneven preparation of the samples as described previously [39].

### Identification and analysis of selected metabolites

HPLC-MS-MS detection of specific compounds was performed on an identical HPLC system with a triple quadrupole mass spectrometer (1200 L, Varian). Chromatography was performed on a polar-modified RP-18 phase and on a HILIC (hydrophilic interaction chromatography) phase. Root exudates and test substances were separated under identical conditions. RP-18 chromatography was conducted as described before [40]. For HILIC, the buffer system consisted of (A) 25 mM ammonium acetate in water and (B) acetonitrile. A 10-μL volume of sample was loaded onto a ZIC-HILIC (Sequant, Haltern am See, Germany) equilibrated with 95% B and separated in a linear gradient from 95-10% B in 10 min. The column was kept at 40°C and the flow rate was 0.2 mL/min. Salicylic acid was identified by the mass transition 137 > 93 [M-H-CO_2_] and a retention time of 7.15 min on the RP-18 phase. Citramalic acid was identified by the mass transition 147 > 87 [M-H-CH_3_COOH] and a retention time of 9.2 min on the HILIC phase.

### P solubility experiment

Solutions of 4-hydroxy benzoic acid (Fluka, Germany), urocanic acid (Fluka, Germany), salicylic acid (Merck, Germany), citramalic acid (Aldrich, Germany), D-arabino-1,4-lactone (Dextera, UK), and pantoic acid (generated from pantolactone purchased from Aldrich, Germany, by hydrolysis in 0.1 M NaOH) were prepared in water at the concentration of 1 mmol L^-1^. pH of the solutions was adjusted to 5.6 with NaOH or HCl. Highly P fixing fossile Oxisol, containing mainly P bound to Al and Fe with 788 mg P kg^-1 ^as Fe/Al-P and 330 mg P kg^-1 ^as Ca-P, clay content 50%, pH(CaCl_2_) 5.6, P concentration in soil solution 0.17 μmol L^-1 ^and Ca acetate-lactate (CAL) extractable P of 4.3 mg kg^-1 ^was fertilized with 100 mg P kg^-1 ^as Ca(H_2_PO_4_)_2_.H_2_O and moistured to 22% w/w water content. After 10 days of equilibration, 9.76 g of moist soil (equivalent to 8.0 g of dry soil) was mixed with 40 mL of the solutions of the compounds listed above or with distilled water. Microbial degradation was prevented by adding two drops of toluene. Each treatment was replicated three times. The samples were shaken on a reciprocal shaker at 150 cycles per minute for 5 h, centrifuged for 15 min at the relative centrifugation force of 2660 g, and the supernatant was filtered though a 0.45-μm nylon membrane filter. Inorganic P concentration was determined by a molybdenum blue colorimetric method [41].

## Authors' contributions

RK performed hydroponic experiments and P solubilization tests. UH and AR performed metabolic profiling and analysis of root exudate components. BS designed and supervised the P solubilization experiment. PK guided metabolic profiling and wrote scripts for data processing. NC conceived the study. All authors evaluated the data, wrote parts of the manuscript and approved the manuscript for submission.

## References

[B1] Bhadoria PBS Steingrobe B Claassen N Leibersbach H Phosphorus efficiency of wheat and sugar beet seedlings grown in soils with mainly calcium, or iron and aluminium phosphate Plant Soil 2002 264 41 52

[B2] Kovar JL Claassen N Sims JT, Sharpley AN Soil-root interactions and phosphorus nutrition of plants Phosphorus: Agriculture and the Environment 2005 Madison, American Society of Agronomy 379 414

[B3] Lynch JM Whipps JM Substrate flow in the rhizosphere Plant Soil 1990 129 1 10 10.1007/BF00011685

[B4] Marschner H Mineral Nutrition of Higher Plants 1995 Academic Press Limited

[B5] Whipps JM Lynch JM Carbon Economy The Rhizosphere 1990 West Sussex. John Wiley and Sons 59 98

[B6] Liebersbach H Steingrobe B Claassen N Roots regulate ion transport in the rhizosphere to counteract reduced mobility in dry soil Plant Soil 2004 260 79 88

[B7] Grimal JY Frossard E Morel JL Maize root mucilage decreases adsorption of phosphate on goethite Biol Fert Soils 2001 33 226 230 10.1007/s003740000312

[B8] Carminati A Moradi AB Vetterlein D Vontobel P Lehmann E Weller U Vogel HJ Oswald SE Dynamics of soil water content in the rhizosphere Plant Soil 2010 332 163 176 10.1007/s11104-010-0283-8 21824150

[B9] Walker TS Bais HP Grotewold E Vivanco JM Root exudation and rhizosphere biology Plant Physiol 2003 132 44 51 10.1104/pp.102.019661 12746510 PMC1540314

[B10] Marschner H Römheld V Strategies of plants for acquisition of iron Plant Soil 1994 165 261 274 10.1007/BF00008069

[B11] Bertin C Yang XH Weston LA The role of root exudates and allelochemicals in the rhizosphere Plant Soil 2003 256 67 83

[B12] Johnson JF Allan DL Vance CP Weiblen G Root carbon dioxide fixation by phosphorus deficient Lupinus albus. contribution to organic acid exudation by proteoid roots Plant Physiol 1996 112 31 41 10.1104/pp.112.1.31 12226371 PMC157919

[B13] Neumann G Römheld V Root excretion of carboxylic acids and protons in phosphorus-deficient plants Plant Soil 1999 211 121 130 10.1023/A:1004380832118

[B14] Hernandez G Ramirez M Valdes-Lopez O Tesfaye M Graham MA Czechowski T Schlereth A Wandrey M Erban A Cheung F Wu HC Lara M Town CD Kopka J Udvardi MK Vance CP Phosphorus stress in common bean: root transcript and metabolic responses Plant Physiol 2007 144 752 767 10.1104/pp.107.096958 17449651 PMC1914166

[B15] Dinkelaker B Römheld V Marschner H Citric acid excretion and precipitation of calcium citrate in the rhizosphere of white lupin (*Lupinus albus L.).* Plant Cell Environ 1989 12 285 292 10.1111/j.1365-3040.1989.tb01942.x

[B16] Gerke J Beißner L Römer W The quantitative effect of chemical phosphate mobilization by carboxylate anions on P uptake by a single root. I. The basic concept and determination of soil parameters J Plant Nutr Soil Sci 2000 163 207 212 10.1002/(SICI)1522-2624(200004)163:2<207::AID-JPLN207>3.0.CO;2-P

[B17] Gardner WK Barber DA Parbery DG The acquisition of phosphorus by *Lupinus albus *L. III. The probable mechanism by which phosphorus movement in the soil/root interface is enhanced Plant Soil 1983 70 107 124 10.1007/BF02374754

[B18] Jones DL Organic acids in the rhizosphere - a critical review Plant Soil 1998 205 25 44 10.1023/A:1004356007312

[B19] Hoffland E Findenegg GR Nelemans JA Solubilization of rock phosphate by rape. II. Local root exudation of organic acids as a response to P starvation Plant Soil 1989 113 161 165 10.1007/BF02280176

[B20] Kania A Neumann G Cesco S Pinton R Römheld V Horst WWJ, Schenk MK, Bürkert A Use of plasma membrane vesicles for examination of phosphorus deficiency-induced root excretion of citrate in cluster root of white lupin (*Lupinus albus *L.) Plant Nutrition-Food Security and Sustainability of Agro-Ecosystems through Basic and Applied Research 2001 Dordrecht, Kluwer Academic Publishers 546 547

[B21] Ae N Arihara J Okada K Yoshihara T Johansen C Phosphorus uptake by pigeon pea and its role in cropping systems of the Indian subcontinent Science 1990 248 477 480 10.1126/science.248.4954.477 17815599

[B22] Reuter JB Edwards DG Wilhelm NS Reuter DJ, Robinson JB Temperate and tropical crops Plant analysis: an interpretation manual 1997 Australia, CSIRO 83 284

[B23] Neumann G Römheld V Pinton R, Varanini Z, Nannipieri P The release of root exudates as affected by plant's physiological status The rhizosphere - biochemistry and organic substances at the soil-plant interface 2001 New York, Marcel Decker, Inc 41 93

[B24] Masoudi-Nejad A Goto S Endo TR Kanehisa M KEGG bioinformatics resource for plant genomics research Methods Mol Biol 2007 406 437 458 18287706 10.1007/978-1-59745-535-0_21

[B25] Hue NV Effects of organic acids/anions on P sorption and phytoavailability in soils with different mineralogies Soil Sci 1991 152 463 471 10.1097/00010694-199112000-00009

[B26] Bolan NS Naidu R Mahimairaja S Baskaran S Influence of low-molecular-weight organic acids on the solubilization of phosphates Biol Fert Soils 1994 18 311 319 10.1007/BF00570634

[B27] Fox TR Comerford NB McFee WW Phosphorus and aluminum release from a spodic horizon mediated by organic acids Soil Sci Soc Am J 1990 54 1763 1767 10.2136/sssaj1990.03615995005400060043x

[B28] Foehse D Jungk A Influence of phosphate and nitrate supply on root hair formation of rape, spinach and tomato plants Plant and Soil 1983 74 359 368 10.1007/BF02181353

[B29] Gahoonia TS Care D Nielsen NE Root hairs and phosphorus acquisition of wheat and barley cultivars Plant and Soil 1997 191 181 188 10.1023/A:1004270201418

[B30] Staunton S Leprince F Effect of pH and some organic anions on the solubility of soil phosphate: implications for P bioavailability Eur J Soil Sci 1996 47 231 239 10.1111/j.1365-2389.1996.tb01394.x

[B31] Xu RK Xiao SC Zhang H Jiang J Ji GL Adsorption of phthalic acid and salicylic acid by two variable charge soils as influenced by sulphate and phosphate Eur J Soil Sci 2007 58 335 342 10.1111/j.1365-2389.2006.00842.x

[B32] Hulme CC The isolation of L-citramalic acid from the peel of apple fruit Biochim Biophys Acta 1954 14 36 43 13160011 10.1016/0006-3002(54)90127-4

[B33] Marconi O Floridi S Montanari L Organic acids profile in tomato juice by HPLC with UV detection J Food Qual 2007 30 253 266 10.1111/j.1745-4557.2007.00119.x

[B34] Fiehn O Kopka J Trethewey RN Wilmitzer L Identification of uncommon plant metabolites based on calculation of elemental compositions using gas chromatography and quadrupole mass spectrometry Anal Chem 2000 72 3573 3580 10.1021/ac991142i 10952545

[B35] Scheffer F Pajenkamp H Phosphatbestimmung in Pflanzenaschen nach der Molibdän-Vanadin-Methode J Plant Nutr Soil Sci 1952 56 2 8

[B36] Tennant D A test of a modified line intersect method of estimating root length J Ecol 1975 63 995 1001 10.2307/2258617

[B37] Ratzinger A Riediger N von Tiedemann A Karlovsky P Salicylic acid and salicylic acid glucoside in xylem sap of *Brassica napus *infected with *Verticillium longisporum* J Plant Res 2009 122 571 579 10.1007/s10265-009-0237-5 19449088 PMC2776162

[B38] Smith CA Want EJ O'Maille G Abagyan R Suizdak G XCMS: processing mass spectrometry data for metabolite profiling using nonlinear peak alignment, matching, and identification Anal Chem 2006 78 779 787 10.1021/ac051437y 16448051

[B39] Laurentin H Ratzinger A Karlovsky P Relationship between metabolic and genomic diversity in sesame (*Sesamum indicum *L.) BMC Genomics 2008 9 250 10.1186/1471-2164-9-250 18510719 PMC2440766

[B40] Adejumo TO Hettwer U Karlovsky P Survey of maize from south-western Nigeria for zearalenone, α- and β-zearalenols, fumonisin B1 and enniatins produced by *Fusarium *species Food Addit Contam 2007 24 993 1000 10.1080/02652030701317285 17691013

[B41] Murphy J Riley JP A modified single solution method for the determination of phosphate in natural waters Anal Chim Acta 1962 27 31 36

